# Removal of Coin Cell Lithium Battery Lodged in the Pediatric Pharyngoesophageal Junction by Rigid Esophagoscopy; a Case Report

**DOI:** 10.22037/aaem.v10i1.1430

**Published:** 2022-01-01

**Authors:** Hisataka Ominato, Takumi Kumai, Yasuaki Harabuchi

**Affiliations:** 1 Department of Otolaryngology-Head & Neck Surgery, Asahikawa Medical University, Asahikawa, Japan.; 2 Department of Innovative Head & Neck Cancer Research and Treatment (IHNCRT), Asahikawa Medical University, Asahikawa, Japan.

**Keywords:** Foreign bodies, esophagoscopes, pediatrics, surgical instruments

## Abstract

A coin cell lithium battery is a common foreign body that can become lodged in the pediatric pharyngoesophageal junction. Because the voltage of such batteries is relatively high, their rapid removal is necessary to avoid mucosal necrosis. Despite being the initial choice for removal, flexible endoscopy cannot remove such foreign bodies from the esophagus. Various removal methods, including rigid esophagoscopy, should be considered for removing lithium coin cell batteries. The transcervical approach is feasible for removing esophageal foreign bodies, but it carries the risk of complications such as esophageal stenosis. Here we report a case of lithium coin battery ingestion that was successfully removed using a rigid esophagoscope. A 2-year-old girl was referred to a local doctor with cough and general fatigue. Chest X-ray and flexible endoscopy revealed a coin cell lithium battery stuck in the pharyngoesophageal junction, but it could not be removed. The foreign body was removed using Nishihata forceps through a rigid esophagoscope under general anesthesia.

## 1. Introduction　

Button batteries are widely used in a variety of household appliances. Since the 1980s, button batteries have become common causes of pediatric foreign bodies in the esophagus (1). Coin cell lithium batteries are 5–25 mm in diameter, making them susceptible to lodging in the pediatric pharyngoesophageal junction (2). Since the voltage of the coin cell lithium battery is 3 V (versus 1.5 V in alkaline batteries) (3), severe tissue damage can be caused by the electric current of the coin cell lithium battery. As sodium hydroxide accumulates on the cathode interface of the coin cell lithium battery due to electrolysis (1, 4), histological changes and damage to the mucosa occur within 15 min and 2 hours of the battery contacting the mucosa, respectively (4). Although flexible endoscopy is often the initial removal method (5), endoscopic forceps cannot effectively remove foreign bodies stuck in the pharyngoesophageal junction. Here we report a case of a coin cell lithium battery foreign body that was difficult to remove using flexible endoscopy, but was successfully removed by rigid esophagoscopy.

## 2. Case presentation

**Figure 1:**
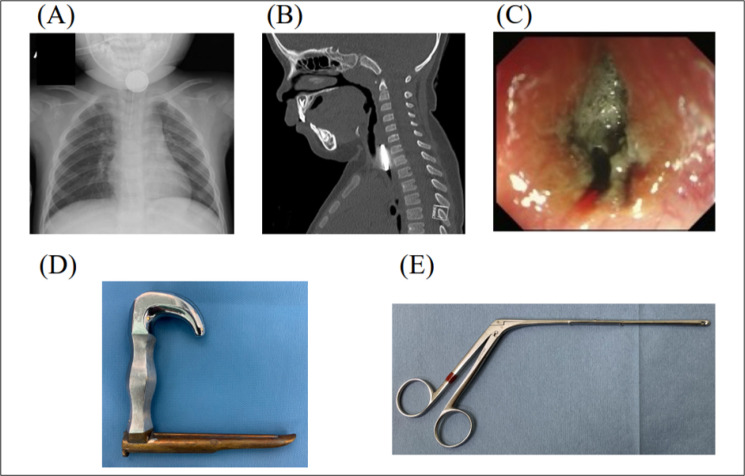
(A) A chest X-ray showing a coin cell lithium battery (double contour) lodged in the upper esophagus. (B) A computed tomography scan showed a coin cell lithium battery lodged in the upper esophagus. (C) Endoscopic findings of the esophagus. The foreign body was stuck in the esophagus and resistant to endoscopic removal. (D) Rigid esophagoscope. (E) Nishihata forceps

**Figure 2:**
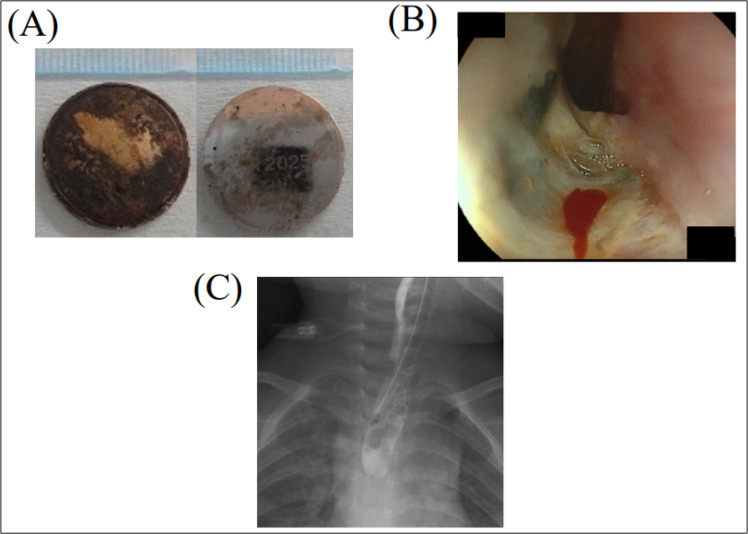
(A) The cathode interface of the coin cell lithium battery was corroded. (B) Flexible endoscopy showed esophageal ulceration without evident perforation. Ulceration was localized to the mucosa where the cathode interface of the coin cell lithium battery was located. (C) Esophagography showed no leakage or stricture

A 2-year-old girl visited a local clinic with general fatigue and cough. The parents described that a coin cell lithium battery was missing at home. A chest X-ray showed a double contour, a typical finding of a coin cell lithium battery, in the upper esophagus (Figure 1A), for which the patient was referred to a secondary referral hospital. Although a coin cell lithium battery was detected in the esophagus using a flexible endoscope, the foreign body had adhered to the mucosa and was immobile, and it could not be removed using endoscopic forceps. Four hours later, the intubated patient was referred to our hospital. A computed tomography scan showed a coin cell lithium battery with a 20 mm diameter in the upper esophagus (Figure 1B). We first attempted to remove the battery using flexible endoscopy. The coin cell lithium battery was tarnished black and had corroded the esophageal mucosa (Figure 1C). Due to adhesion, the foreign body could not be removed endoscopically. Next, we performed rigid esophagoscopy to remove the battery. Opening the pharyngoesophageal junction using a rigid esophagoscope revealed that part of the esophageal mucosa attached to the cathode interface of the coin cell lithium battery was ulcerated. Although the foreign body was attached to the mucosa, it was successfully removed using Nishihata forceps under rigid esophagoscopy (Figures 1D, 1E). No mucosal perforation was evident. The cathode interface of the removed battery was corroded (Figure 2A). The suspected duration of the battery–esophagus adhesion was at least 12 h.The patient was kept intubated for 10 days after removal of the battery, and treated with antibiotics (ampicillin-sulbactam) to rest the esophageal mucosa. Flexible endoscopy and esophagography showed esophageal ulceration without perforation, leakage, or stricture on postoperative day 10 (Figures 2B, 2C). She was discharged on postoperative day 26 without any problems with oral intake. No evidence of esophageal stricture was detected during follow-up.

## 3. Discussion

A coin cell lithium battery is thin (1–6 mm thick) and has a diameter of approximately 20 mm. If accidentally swallowed, the battery is likely to stagnate at physiological constrictions of the esophagus including the pharyngoesophageal junction (2). The high voltage of the coin cell lithium battery induces more severe tissue damage compared to an alkaline battery (3). Within 2 h, hydroxide ions generated on the cathode side of the battery through electrolysis damage the esophageal mucosa (4). Recent epidemiological studies in the United States have reported that the number of accidents and serious complications involving the ingestion of batteries has been increasing, especially since the advent of lithium batteries (6). In detection of a coin cell lithium battery as a foreign body, the double contour sign is a characteristic finding on chest X-ray (7). Complications associated with coin cell lithium battery ingestion include ulceration, esophageal stenosis, esophageal tracheal fistula, esophageal perforation, large vessel injury, mediastinitis, and recurrent nerve palsy (8). Since corrosion is stronger on the cathode side of the battery, the anatomical region it contacts should be carefully examined to predict possible complications (9). Immediate removal of the battery is required to avoid serious complications. Flexible endoscopy, rigid esophagoscopy, and external cervical incisions are commonly used for esophageal foreign body removal (5). After removal, fasting and antibacterial drugs are prescribed to avoid infection and/or perforation through the mucosal ulcers. The use of steroids to prevent stricture after esophageal ulceration has been reported for alkaline burns; however, further investigations are required (10).

Flexible endoscopy is currently the minimally invasive approach of choice for removing foreign bodies in the upper digestive tract (5). However, the grasping power of tools applied in flexible endoscopy is weak, and endoscopic removal is not possible in cases of solid adhesions of the foreign body. In our case, flexible endoscopy, rigid esophagoscopy, and a transcervical incision kit were prepared, and the coin cell lithium battery was removed by rigid esophagoscopy within 1 h after the patient’s arrival. To avoid surgery-related complications, a transcervical incision should be considered the last option. The safety of rigid esophagoscopy is comparable to that of flexible endoscopy (5). The handling of a foreign body using forceps through rigid esophagoscopy is easier than when using endoscopic forceps, which has a weak grasping power. In this case, Nishihata forceps, which are used in endoscopic sinus surgery, had suitable length and grasping power to remove the stuck battery from the pharyngoesophageal junction. The direct handling of foreign bodies by rigid esophagoscopy is a safe and effective approach for removing pediatric foreign bodies from the esophagus when flexible endoscopy is inapplicable.

## 4. Conclusion

Although rigid esophagoscopy is a classic method, it is efficient for removing stuck foreign bodies from the pharyngoesophageal junction. Various removal approaches should be considered to remove lithium coin cell batteries, which can induce severe mucosal damage.

## 5. Declarations

### 5.1 Acknowledgments

We would like to thank Editage (www.editage.com) for English language editing.

### 5.2 Authors' contributions

All authors met the criteria for authorship contribution based on recommendations of international committee of medical journal editors. HO and TK wrote the manuscript. YH helped shape the manuscript.

### 5.3 Funding and support

None.

### 5.4 Conflict of interest

Authors have no conflict of interest.
